# Systematics of Lobelioideae (Campanulaceae): review, phylogenetic and biogeographic analyses

**DOI:** 10.3897/phytokeys.174.59555

**Published:** 2021-03-05

**Authors:** Samuel Paul Kagame, Andrew W. Gichira, Ling-Yun Chen, Qing-Feng Wang

**Affiliations:** 1 Key Laboratory of Plant Germplasm Enhancement and Specialty Agriculture, Wuhan Botanical Garden, Chinese Academy of Sciences, Wuhan 430074, China; 2 University of Chinese Academy of Sciences, Beijing 100049, China; 3 Sino-Africa Joint Research Center, Chinese Academy of Sciences, Wuhan 430074, China; 4 State Key Laboratory of Natural Medicines, Jiangsu Key Laboratory of TCM Evaluation and Translational Research, School of Traditional Chinese Pharmacy, China Pharmaceutical University, Nanjing 211198, China

**Keywords:** Lobelioideae, monophyletic, polyphyletic

## Abstract

Lobelioideae, the largest subfamily within Campanulaceae, includes 33 genera and approximately1200 species. It is characterized by resupinate flowers with zygomorphic corollas and connate anthers and is widely distributed across the world. The systematics of Lobelioideae has been quite challenging over the years, with different scholars postulating varying theories. To outline major progress and highlight the existing systematic problems in Lobelioideae, we conducted a literature review on this subfamily. Additionally, we conducted phylogenetic and biogeographic analyses for Lobelioideae using plastids and internal transcribed spacer regions. We found that former studies have reached agreement on the southern African origin of Lobelioideae, herbaceous habit and Asian origin of giant lobelioids, the convergent evolution of giant rosette lobelioids, and lastly, the multiple cosmopolitan and independent radiation of lobelioids in Africa, Pacific Basin, and the Hawaiian Islands. Also, *Apetahia* Baill., *Sclerotheca* A.DC., and *Cyanea* Gaudich. are paraphyletic, while *Lobelia* L., *Pratia* Gaudich., *Centropogon* C.Presl, *Siphocampylus* Pohl, and *Isotoma* Lindl. are polyphyletic. The taxonomy of these genera, especially *Lobelia*, is particularly quite frustrating. This calls for further reappraisals using both morphological and molecular data.

## Introduction

Lobelioideae, the largest subfamily within Campanulaceae, includes 31 genera and approximately 1200 species (Knox et al. 2008a). They are characterized by resupinate flowers with zygomorphic corollas and connate anthers (Lammers 2011). They are widely distributed across the world, but absent in the Middle East, Arctic, and some sections of the Central Asia region, with half of them native to South America (Lagomarsino et al. 2014). Some species, such as *Lobelia siphilitica* L. (Linnaeus 1753), *Lobelia erinus* L. (Linnaeus 1753), and *Lobelia cardinalis* L. (Linnaeus 1753), are known for their horticultural value (Lagomarsino et al. 2014). Approximately half of the species in this subfamily belong to three Neotropical genera: *Centropogon* C.Presl, (Presl 1836) (approximately 210 species), distributed from southern Mexico to Bolivia and Brazil, with two species in the lesser Antilles; *Burmeistera* H.Karst. and Triana (Karsten and Triana 1857), (approximately 100 species) distributed in Guatemala through northern Peru; and *Siphocampylus* Pohl (Pohl 1827), (approximately 230 species) distributed from Costa Rica to Argentina and Greater Antilles (Lammers 2007a).

Since the 1990s, many researchers have studied the systematics and biogeography of Lobelioideae using morphological and molecular data, for example, Lammers (1990, 1991, 1993), Knox and Kowal (1993), Knox et al. (1993), Lammers et al. (1993), Givnish et al. (1994, 2009, 2013), Givnish (1995, 1998, 2010), Antonelli (2009), Lagomarsino et al. (2014), Chen et al. (2016), and Knox and Li (2017), among others. However, the systematics of Lobelioideae has been full of contradictory conclusions. Almost all recent classifications involving this subfamily, for example, Lammers (1990, 1991, 1993, 2011), Knox and Kowal (1993), Givnish et al. (1994, 2009, 2013), Givnish (1995, 1998, 2010), Antonelli (2009), Lagomarsino et al. (2014), Chen et al. (2016), and Knox and Li (2017), among others, contradict early taxonomic conclusions of Wimmer (1943, 1953, 1968), McVaugh (1949a), and Murata (1995). For instance, Lammers (2011) recommended the need for revision in the genus *Lobelia* L. (Linnaeus 1753). Lammers (2011) claimed that Wimmer (1943,^1953^), based his classification on a few morphological characters. He also added that Murata (1995) only stated the exemplars for each taxon instead of assigning species to their taxonomic groups. Additionally, both Wimmer (1943,^1953^) and Murata (1995) violated the International Code of Botanical Nomenclature (ICBN) with their classification (Lammers 2011).

Given these recent studies, there is an emerging need to outline the major progress and the existing systematic and biogeographic problems in the Lobelioideae subfamily. To meet this need, we conducted a literature review, phylogenetic, and biogeographic analyses of this subfamily using almost all available sequences of family Campanulaceae from the GenBank.

## Materials and methods

### Literature sources

The systematics of Lobelioideae was explored by checking literature works through online libraries and journals. We explored previous works to understand the debates and contentions that had been there previously and the steps that had been taken to solve the contentions. We also wanted to have a general overview of the taxonomic progress with regards to this subfamily (Zuccarini 1832; McVaugh 1941, 1943a, b, 1949a, b, 1955; Wimmer 1943, 1953, 1968; Bowden 1959, 1961; Moeliono and Tuyn 1960; Carlquist 1962, 1969, 1974; Carlquist et al. 1965; Mabberley 1974, 1975; Thulin 1986a, 1991; Thulin et al. 1986b; Phillipson 1989; Ayers 1990; Lammers 1990, 1991, 1993, 2007a, b, 2010, 2011; Murray and Cameron 1990; Harvey 1992; Lammers and Hensold 1992; Lammers et al. 1993; Knox and Kowal 1993; Knox et al. 1993; Cosner et al. 1994; Givnish et al. 1994; Givnish 1995, 1998; Gustaffson and Bremer 1995; Murata 1995; Vieira and Shepherd 1998; Serra et al. 1999; Givnish 2000; Schultheis 2001a, b; Eddie et al. 2003; Givnish et al. 2004; Knox et al. 2004, 2008a, b; Murray et al. 2004; Knox 2005, 2014; Koopman and Ayers 2005; Knox et al. 2006; Antonelli 2008, 2009; Givnish et al. 2009; Haberle et al. 2009; Givnish 2010; Givnish et al. 2013; Crowl et al. 2014; Lagomarsino et al. 2014, 2016; Chen et al. 2016; Crowl et al. 2016; Knox and Li 2017; Uribe-Convers et al. 2017; Hunter 2018).

### Taxon sampling

We aimed to include as many of the Lobelioideae species as possible. Nineteen loci were obtained, that is, eighteen plastid gene loci (*atpB*-*rbcL* spacer, *atpB*, *atpF*, *atpF*-*atpH* spacer, *atpH*, *matK*, *ndhF*, *psbA*-*trnH* spacer, *psbA*-*trnK* spacer, *petD*, *rbcL*, *rpoC1*, *trnL*-*trnF* spacer, *trnT*-*trnL* spacer, *trnV*-*trnK* spacer, *trnK*-*matK* spacer, *rpl32*-*ndhF* spacer, *rpl16*) and one nuclear gene, internal transcribed spacer (ITS). These sequences were generated using the NCBI ENTREZ UTILITY (Kans 2020) program (Accessed 1^ST^ April 2020) and double-checked manually at the GenBank database. Additionally, almost all available Campanulaceae complete plastid genomes were manually accessed from the GenBank. The respective plastid regions were extracted using NCBI BLASTN v. 2.9.0+ (Camacho 2018) with default settings. We included nine outgroup taxa to increase the chances of recovering the early branching of Campanulaceae (Knox 2014). The outgroups included: *Abrophyllum ornans* (F.Muell.) Benth. (Bentham and Mueller 1864), *Carpodetus serratus* J.R.Forst. & G.Forst. (Forster 1776), *Corokia cotoneaster* Raoul (Raoul 1846), *Cuttsia viburnea* F.Muell. (Mueller 1865), *Pentaphragma ellipticum* Poulsen (Poulsen 1903), *Phelline lucida* Vieill. ex Bail. (Baillon 1872), *Roussea simplex* Sm. (Smith 1789), *Scaevola* sp. L. (Linnaeus 1771) and *Stylidium adnatum* R.Br. (Brown 1810). Taxa voucher information and GenBank accession numbers are provided in the Suppl. material 1: Lobelioideae data matrix.

### Alignment and phylogenetic analyses

All the gene regions were aligned separately using MAFFT v. 7.429 (Katoh and Standley 2013) with an adjust-direction and 1000 maximum iterations options. The alignment of each region was manually checked and taxa with short sequences (≤ 200bp) were edited using GENEIOUS Pro v. 5.6.4 (Kearse et al. 2012). Edited sequences were analyzed using PHYUTILITY v. 2.2.6 (Smith and Dunn 2008) to delete gaps and ambiguous sequences. The indels within the sequences were treated as missing data and they were therefore excluded from the analysis. The *trnF*-*trnL* spacer region had the highest number of sequences while *atpF* recorded the least (Table 1). Each dataset was analyzed using JMODELTEST v. 2.1.10 (Darriba et al. 2012) to determine the best evolution substitution model (Table 1). Maximum Likelihood (ML) analysis for each of the aligned dataset was done using RAxML v. 8.2.12 (Stamatakis 2014). Datasets with unavailable models were analyzed using the GTRCAT model. Each dataset was analyzed using 100 bootstrap values to measure clade support. After pilot phylogenetic analyses, nine plastid regions, that is, *atpB*, *atpF*, *atpF*-*atpH* spacer, *atpH*, *matK*, *psbA*-*trnK* spacer, *petD*, *rbcL*, and *rpoc1*, were selected (Table 1) as they had a better phylogenetic resolution. The nine plastid regions were concatenated to form a combined plastid (cp) dataset and used for ML analyses with the best-selected model. The selection of the best substitution model of the combined dataset did not employ the use of partitioning in this analysis. ITS region was also subjected to ML analyses separately since it formed a tree with a poor resolution when combined with the plastid regions.

**Table 1.** Gene regions used in this study.

Dataset	Gene region	#Seq.	Total seq. length (bp)	Aligned seq. length (bp)	Models
*Plastid region*	*atpB* ^†^	453	1,402	1,334	GTR+I+G
	*atpB-rbcL*	350	809	643	TVM+I+G
	*atpF* ^†^	126	375	360	GTR+G
	*atpF-atpH* ^†^	169	605	529	GTR+I+G
	*atpH* ^†^	134	243	235	TVM+I+G
	*matK* ^†^	466	872	781	TVM+I+G
	*ndhF*	153	2,177	2,002	GTR+I+G
	*psbA-trnH*	279	367	263	GTR+I+G
	*psbA-trnK* ^†^	136	1,264	1,219	GTR+I+G
	*petD* ^†^	696	889	818	TVM+I+G
	*rbcL* ^†^	681	1,131	1,076	TVM+I+G
	*rpoc1* ^†^	187	621	596	TVM+I+G
	*trnL-trnF*	701	875	743	GTR+I+G
	*trnT-trnL*	127	1,191	1,114	TVM+I+G
	*trnV-trnK*	173	654	600	TVM+I+G
	*trnK-matK*	374	2361	2,155	TVM+I+G
	*rpl32-ndhF*	250	698	587	TVM+G
	*rpl16*	402	901	791	GTR+I+G
	Combined	991	7,402	4,826	TMV+I+G
*Nuclear*	*ITS*	642	669	471	GTR+I+G

† = gene regions that were concatenated to form combined plastid (cp) dataset. **Seq.** = Sequences. #**Seq.** = Total number of sequences (including outgroups).

### Biogeography analyses

Biogeographic analyses were conducted in MESQUITE v. 3.61 (Maddison and Maddison 2019) using the parsimony ancestral states reconstruction method. The biogeographic regions were divided into Africa (Madagascar, tropical, and southern Africa), America (North, Central, and South America), Asia (tropical and temperate Asia), Australasia (Australia and New Zealand), Mediterranean (northern Africa, Cyprus, Sicily, Sardinia, and Crete) and the Pacific Islands (Hawaii, Kaua’i, French Polynesia, Rarotonga, and the Marquesas Islands) according to Chen et al. (2016). The ancestral regions for the outgroups species, Campanulaceae sp. and *Lobelia* sp. were unclear and therefore were not assigned any value (region), however, the reconstruction method employed was set to consider missing and inapplicable data.

### Data resources

The data underpinning the analysis reported in this paper are deposited in the Dryad Data Repository at https://doi.org/10.5061/dryad.3xsj3txfw.

## Results

We accessed eighteen plastid loci and one nuclear gene region of almost all available Campanulaceae species, out of which, nine plastid regions were selected for the combined plastid region datasets. The combined plastid (cp) region dataset included 981 Campanulaceae species, with 298 species from Lobelioideae, which covered almost all Lobelioideae species available in GenBank (Accessed on 1^st^ April 2020) (Table 2).

**Table 2.** List of genera used in this study.

Genus	No. of accepted species	No. of species in this study	References
*Apetahia* Bail.	4	3	(Baillon 1882)
*Brighamia* A.Gray	2	1	(Gray 1867)
*Burmeistera* H.Karst. and Triana	103	28	(Karsten and Triana 1857)
*Centropogon* C.Presl	215	41	(Presl 1836)
*Clermontia* Gaudich.	33	18	(Gaudichaud-Beaupré 1829)
*Cyanea* Gaudich.	85	6	(Gaudichaud-Beaupré 1829)
*Delissea* Gaudich.	16	1	(Gaudichaud-Beaupré 1829)
*Dialypetalum* Benth.	6	2	(Bentham 1876)
*Diastatea* Scheidw.	7	1	(Scheidweiler 1841)
*Downingia* Torr.	15	5	(Torrey 1857)
*Grammatotheca* C.Presl	1	1	(Presl 1836)
*Hippobroma* G.Don	1	1	(Don 1834)
*Hypsela* C.Presl	–	1	(Presl 1836)
*Isotoma* Lindl.	13	7	(Lindley 1826)
*Legenere* McVaugh	1	2	(McVaugh 1943a)
*Lithotoma* E.B.Knox	–	1	(Knox 2014)
*Lobelia* L.	437	117	(Linnaeus 1753)
*Lysipomia* Kunth	35	3	(Kunth 1818)
*Monopsis* Salisb.	18	5	(Salisbury 1817)
*Palmerella* A.Gray	2	1	(Gray 1876)
*Porterella* Torr.	1	1	(Hayden 1872)
*Pratia* Gaudich.	–	4	(Gaudichaud-Beaupré 1829)
*Sclerotheca* A.DC.	6	8	(Candolle 1839)
*Siphocampylus* Pohl	235	32	(Pohl 1827)
*Solenopsis* C.Presl	7	4	(Presl 1836)
*Trematolobelia* Zahlbr. ex Rock	8	2	(Rock 1913)
*Wimmerella* Serra, M.B.Crespo and Lammers	10	2	(Serra et al 1999)

– No accepted species available only synonyms.

**Figure 1.**
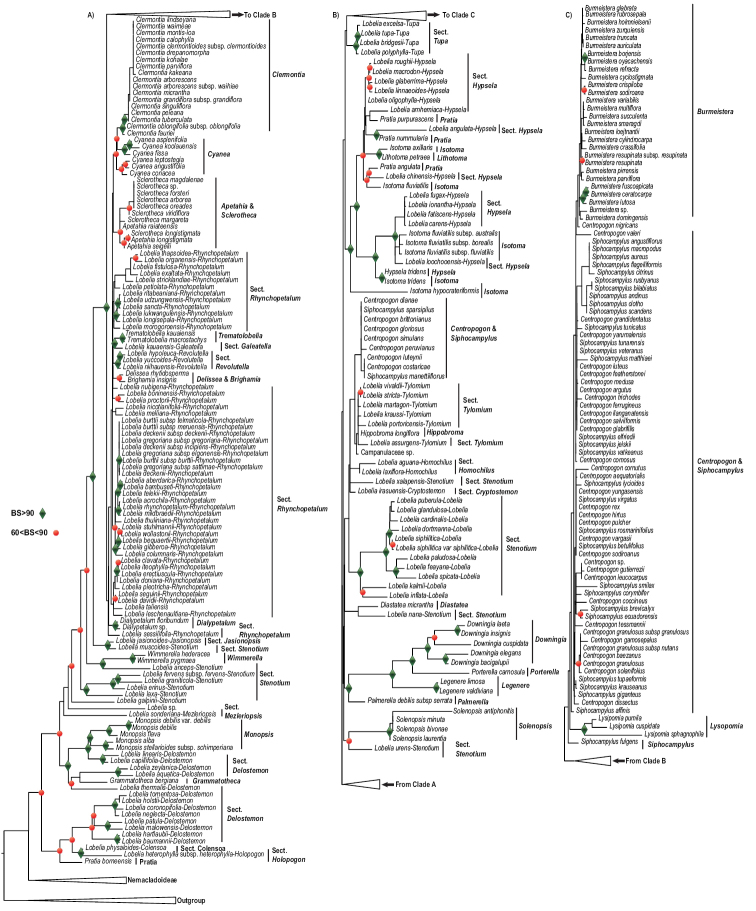
Phylogeny, genera, and bootstrap values of Lobelioideae using combined plastid (cp) regions dataset.

The combined plastid dataset had representatives from all genera except *Howellia* A.Gray (Gray 1879), *Heterotoma* Zucc. (Zuccarini 1832), *Ruthiella* Steenis (van Steenis 1965), *Dielsantha* E.Wimm. (Wimmer 1948), *Trimeris* C.Presl (Presl 1836), and *Unigenes* E.Wimm. (Wimmer 1948) (Table 3). The interspecific bootstrap (BS) values were quite distinct. The BS value for the *Clermontia*, *Centropogon*, *Burmeistera*, and *Siphocampylus* clades recorded the least BS values. The combined plastid (cp) dataset showcased a better phylogram with a higher sampled taxon and a clearer resolution (Fig. 1) than the nuclear gene phylogeny.

**Table 3.** Classification and biogeography of Lobelioideae (Campanulaceae).

Genus	Sections (§)	No. of Species		Monophyletic				Ancestral region		
(Crowl 2016)	(Lammers 2011)	(Lammers 2011)	Current	Lammers (2011)	Antonelli (2008)	Chen (2016)	Current	Lammers (2011)	Chen (2016)	Current
* Lobelia *	* Holopogon *	14	1	Yes	No	–	–	Australia	Australasia	Australasia
	* Colensoa *	1	1	–	No	–	–	New Zealand	Australasia	Australasia
	* Delostemon *	44	14	Yes	–	No	No	S. Africa, T. Africa, S.E Asia	Africa	Africa, Asia, America
	* Mezleriopsis *	7	1	Yes	No	–	–	Africa	Africa	Africa
	* Stenotium *	144	10	Yes	Yes	No	No	Africa (Tropical and Southern), Med, America (North and South), S.E Asia	Africa, Med, America	Africa, Med, America
	* Jasionopsis *	1	1	Yes	–	–	–	Africa	Africa	Africa
	* Rhynchopetalum *	61	48	Yes	No	No	No	S.E Asia, T. Africa, S. America, Asian Islands	Asia, P. Islands, Africa, America	Asia, America, Africa
	* Revolutella *	9	3	Yes	–	Yes	Yes (H)	Hawaii	P. Islands	P. Islands
	* Lobelia *	22	11	Yes	No	No	Yes (H)	N. America	America	America
	* Cryptostemon *	9	1	Yes	–	Yes	–	America	America	America
	* Homochilus *	5	2	Yes	Yes	–	Yes (L)	America	America	America
	* Tylomium *	38	6	Yes	–	No	No	N. America	America	America
	* Hypsela *	43	13	Yes	No	No	No	S.E Asia, Australasia, S. America	America, Australasia, Asian Islands, Asia	Australasia, Asia, America
	* Tupa *	4	4	Yes	Yes	Yes	Yes (H)	S. America	America	America
	* Galeatella *	5	1	Yes	–	Yes	–	Hawaii	–	–
	* Plagiobotrys *	1	–	Yes	–	–	–	Malesia	–	–
	* Trimeris *	1	–	Yes	–	–	–	St. Helena	–	–
	* Speirema *	5	–	Yes	–	–	–	S.E Asia	–	
* Pratia *	–	13	4	–	–	–	No	–	–	Australasia
* Grammatotheca *	–	1	1	–	–	–	–	–	Africa	Africa
* Monopsis *	–	20	5	–	–	Yes	Yes (H)	–	Africa	Africa
* Wimmerella *	–	10	2	–	–	–	Yes (H)	–	Africa	Africa
* Dialypetalum *	–	6	2	–	–	–	Yes (H)	–	–	Africa
* Delissea *	–	10	1	–	–	Yes	–	–	P. Islands	P. Islands
* Brighamia *	–	2	1	–	–	Yes	–	–	P. Islands	P. Islands
* Trematolobelia *	–	4	2	–	–	Yes	Yes (H)	–	P. Islands	P. Islands
* Apetahia *	–	4	2			No	No	–	P. Islands	P. Islands
* Sclerotheca *	–	10	8	–	–	Yes	No	–	P. Islands	P. Islands
* Cyanea *	–	70	6	–	Yes	No	Yes (H)	–	P. Islands	P. Islands
* Clermontia *	–	22	18	–	–	No	Yes (H)	–	P. Islands	P. Islands
* Solenopsis *	–	10	4	–	–	Yes	Yes (H)	–	Europe	Mediterranean
* Downingia *	–	13	5	–	Yes	Yes	Yes (H)	–	America	America
* Legenere *	–	2	2	–	–	–	Yes (H)	–	America	America
* Palmerella *	–	2	1	–	–	–	–	–	America	America
* Porterella *	–	1	1	–	–	–	–	–	America	America
* Diastatea *	–	6	1	–	–	–	–	–	America	America
* Hippobroma *	–	1	1	–	–	–	–	–	America	America
* Isotoma *	–	12	7	–	–	No	No	–	Australasia	Australasia
* Hypsela *	–	1	1	–	–	–	–	–	–	Australasia
* Lithotoma *	–	1	1	–	–	–	–	–	–	Australasia
* Lysipomia *	–	40	3	–	Yes	Yes	Yes (H)	–	America	America
* Siphocampylus *	–	220	32	–	Yes	No	No	–	America	America
* Burmeistera *	–	102	28	–	Yes	Yes	Yes (L)	–	America	America
* Centropogon *	–	49	41	–	Yes	No	No	–	America	America
* Howellia *	–	1	–	–	–	–	–	–	America	–
* Heterotoma *	–	1	–	–	–	–	–	–	–	–
* Ruthiella *	–	4	–	–	–	–	–	–	–	–
* Trimeris *	–	1	–	–	–	–	–	–	–	–
* Unigenes *	–	1	–	–	–	–	–	–	–	–
* Dielsantha *	–	1	–	–	–	–	–	–	–	–

–, unknown or uncertain. L = Bootstrap value <60. M = 60 ≤ BS < 90. H = BS ≥90. P. Islands = Pacific Islands (Hawaii, French Polynesia, Rarotonga, and the Marquesas Islands). Med = Mediterranean (N. Africa, Cyprus, Sicily, Sardinia, and Crete). Australasia = (Australia and N. Zealand). Asia = (Tropical and Temperate Asia). Africa = (Madagascar, Tropical, and S. Africa). America = (North, Central, and South America).

## Discussion

### Agreements on previous debates

Many scholars have expressed their insights with the existing systematics of the Lobelioideae genera (Lammers 2007a; Givnish 2010; Chen et al. 2016; Knox and Li 2017). The uncertainty in circumscription among different lineages in Lobelioideae has been a result of rapid diversification and divergence of this subfamily approximately 20 million years ago (Knox and Li 2017). After extensive literature search and reviews, we found three main areas that were previously in contention: South African origin of Lobelioideae (Mabberley 1975; Knox and Li 2017), herbaceous habit, and Asian origin of giant lobelioids (Carlquist 1962; Mabberley 1974; Chen et al. 2016), and the convergent evolution of giant rosette (perennial monocarpic herbs mostly occurring in alpine and subalpine bogs) lobelioids (Antonelli 2009; Givnish 2010). Currently, agreements regarding these contentions appear to have been reached and are in accord with our analyses.

The geographical origin of the Lobelioideae had been a point of contention, with different scholars having varying biogeographic theories. Mabberley (1975) suggested a South American origin of lobelioids. Mabberley (1975) postulated that the South American pachycaul lobelioids gave rise to plants, which spread to Chile and the Caribbean (*Lobelia* § *Tylomium* (C.Presl) Benth. (Bentham 1876)), Hawaii (*Trematolobelia*) and Brazil (*Lobelia* § *Rhynchopetalum* (Fresen.) Benth. (Bentham 1876)). He added that the rise of winged seeds in Hawaii permitted the inter-island spread of lobelioids and in Brazil, it allowed the *Lobelia* § *Rhynchopetalum* to travel to Africa. However, Knox et al. (1993, 2008a) stated that the South American species are mixed assemblage, possibly involving pantropical dispersal events. Knox et al. (2006), Chen et al. (2016), and Knox and Li (2017) concluded Lobelioideae originated from South Africa and underwent multiple cosmopolitan radiation events. Our results supported the ‘Out of Africa’ hypothesis and multiple cosmopolitan radiations of Lobelioideae, which corroborated Antonelli (2009), Chen et al. (2016), and Knox and Li (2017).

The ancestral habit type and origin of the giant Lobelioids have been in the limelight for years. Carlquist (1962, 1969), using wood anatomy, suggested an herbaceous origin of giant lobelioids. However, Mabberley (1974, 1975) challenged the above sentiment and suggested that the herbaceous species of lobelioids have been derived from large, thick-stemmed ancestors (*Lobelia* § *Rhynchopetalum* and *Lobelia* § *Tylomium*). He added that the herbaceous habit of lobelioids is an advanced character. Knox et al. (1993), using cpDNA restriction sites and inversions, supported Carlquist’s (1962) hypotheses of herbaceous ancestry. Givnish (2000), based on molecular phylogenetic analysis, showed that the ancestor of the Hawaiian lobelioids was most likely woody, corroborating Mabberley’s (1974, 1975) proposals. Also, Givnish et al. (2009) hypothesized that an Asian group – represented by the placeholder *Lobelia nicotianifolia* Roth (Roth 1821) – might have the ancestral stock from which both Pacific and African giant lobelioids had evolved. Most recently, Chen et al. (2016) confirmed (1) the herbaceous habit of lobelioids ancestors, and (2) the Asian origin of giant lobelioids. Knox and Li (2017) corroborated Chen et al. (2016) and added that extant Hawaiian/Pacific and Brazilian/African giant lobelioids are derived from herbaceous giant lobelioids (Knox and Li 2017). Our results corroborated that of Chen et al. (2016) and Knox and Li (2017), in which, herbaceous ancestry of giant rosette lobelioids was well illustrated.

The Hawaiian lobelioids form a remarkable clade, encompassing more species than any other plant clade restricted to a single oceanic island or archipelago, and their geographic source has been hotly debated (Givnish et al. 1994, 2009; Givnish 1995). They have long been viewed as one of the most spectacular cases of adaptive radiation in plants on oceanic islands (Carlquist et al. 1965; Carlquist 1974; Lammers 1990; Givnish et al. 1994, 2004; Givnish 1995; Givnish 1998; Givnish and Montgomery 2014). Wimmer (1953) and Mabberley (1974, 1975) postulated that fleshy-fruited genera are a product of a single colonization event while capsular-fruited taxa are products of more than one colonization event. However, Givnish (2000), using molecular phylogenetic analysis, illustrated that Hawaiian lobelioids are instead a product of a single immigration event. Antonelli (2009) suggested that the Hawaiian and African giant lobelioids appeared to have evolved from a single common ancestor. However, Givnish (2010) refuted those claims and argued that the giant rosette lobelioids are an exemplar of convergent evolution rather than single common ancestry. Our analysis corroborated one of Givnish’s (2010) illustrations on lobelioids’ convergent evolution theory, that is, compared to the rest of lobelioids species, only a minority number of species (*Lobelia* § *Rhynchopetalum*) have the giant rosette growth form adapted to alpine or mountain conditions, with non-rosette species forming the remainder of the clade. These ‘rosette-species’ are embedded within the non-rosette species (Fig. 1), a clear indication that indeed the giant rosette lobelioids are a result of convergent evolution. Knox and Li (2017) also used maximum-likelihood analyses of whole plastomes to conclude that the giant African lobelioids (including some descendants in South America) were sister to the Pacific giant lobeliads as a whole and with *Lobelia boninensis* Koidz. (Nakai 1920), from the Bonin Islands, then *Apetahia*/*Sclerotheca* from the Society Islands and the Cook Islands forming a sister to the Hawaiian lobelioids, and then all of them forming a sister to some Asian giant lobelioids, corroborating the proposal by Givnish et al. (2004). Our combined plastid data ties together *Delissea*-*Brighamia*, *Trematolobelia*, *Lobelia* § *Galeatella*, and *Lobelia* § *Revolutella*, all from Hawaii, closely related to giant African and South American lobelioids.

In addition, Knox and Li (2017) summarized the cosmopolitan radiation of lobelioids in four out-of-Africa dispersal scenarios. (1) The biogeographic pattern of South African species relative to lobelioids elsewhere in the world maps Lobelioideae ancestry to the modern-day Western Cape Province (Knox and Li 2017). *Lobelia anceps* L.f. (Linnaeus 1782), for instance, originated from South Africa and subsequently dispersed to many other southern hemisphere sites, including New Zealand (Knox et al. 2006). Madagascar acted as a stepping-stone to eastern Asia where the robust, herbaceous, hemicryptophyte growth form evolved from (Knox and Li 2017). (2) The Amphi-tropical dispersal from the Western Cape to the Mediterranean region did occur and with rapid subsequent dispersal to the North America region (Knox and Li 2017). (3) The dispersal from South Africa to South America stood the greatest likelihood of success if the initial colonization occurred at a similar latitude with a similar habitat (Knox and Li 2017). The circumscription of *Lobelia xongorolana* E.Wimm. (Wimmer 1935) (Endemic in Angola) as the sister lineage to the Brazilian species (Mabberley 1974), would implicate Angola as a stepping-stone in dispersal to Brazil, whereas a true sister-species relationship with *Lobelia stricklandiae* would suggest that dispersal to Brazil originated from East Africa and that dispersal to Angola was a separate event (Knox and Li 2017). (4) Successful colonization of Australia from South Africa also would have been favored by latitudinal and habitat similarity. The Western Australia endemic *Isotoma hypocrateriformis* Druce (Druce 1917), is sister to the remaining species in this predominantly Australasian clade that subsequently diversified in most Australian habitats, dispersed on three separate occasions to New Zealand (Knox et al. 2008b; Heenan et al. 2008), and dispersed twice to eastern Asia (Knox and Li 2017).

### Phylogeny and biogeography of lobelioideae

**Figure 2.**
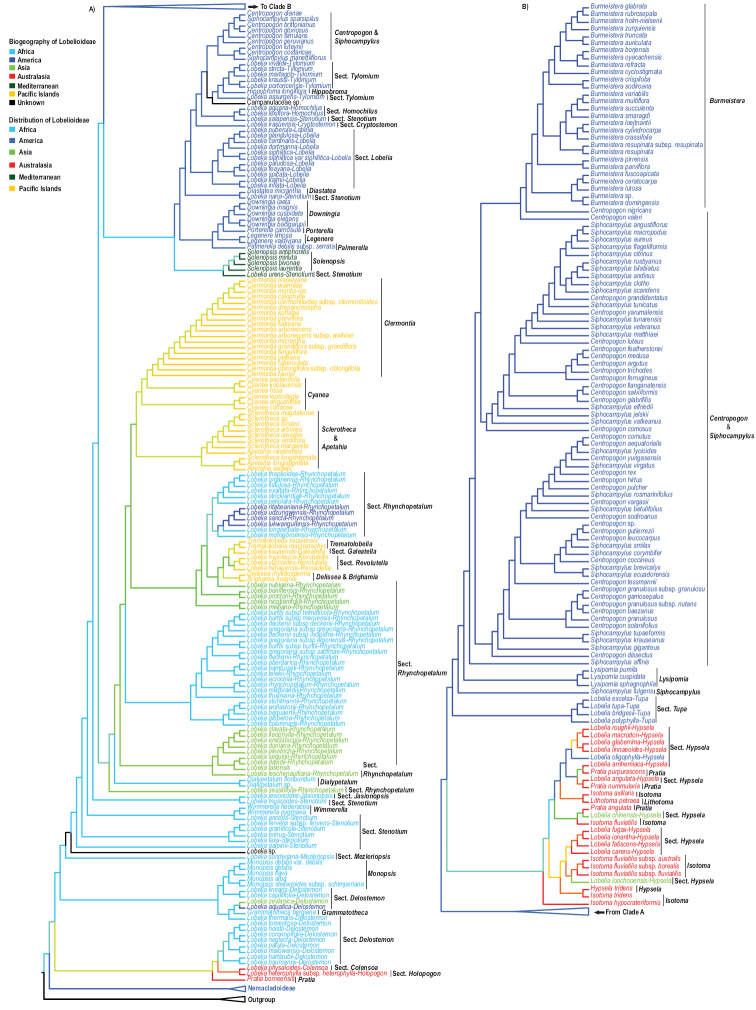
Biogeography of Lobelioideae combined plastid (cp) region datasets using parsimony ancestral state reconstruction. Taxa color coding represents the geographical distribution of the species.

Lobelioideae consisted of up to 31 genera (Knox et al. 2008a). However, through our extensive literature review, we found a total of 33 currently documented genera (Table 3). We sampled 27 out of the 33 Lobelioideae genera in our combined plastid (cp) dataset (for easy understanding, subsequent discussion part is based on the combined (cp) plastid tree (Fig. 1; Fig. 2) and the ITS region tree. Our analyses found ten monophyletic Lobelioideae genera, that is, *Monopsis*, *Wimmerella*, *Dialypetalum*, *Clermontia*, *Solenopsis*, *Legenere*, *Downingia*, *Burmeistera*, *Lysipomia*, and *Trematolobelia*, three paraphyletic genera, that is, *Apetahia*, *Sclerotheca*, and *Cyanea*, and lastly, five polyphyletic genera, that is, *Lobelia*, *Pratia*, *Centropogon*, *Siphocampylus*, and *Isotoma*. *Grammatotheca*, *Delissea*, *Brighamia*, *Palmerella*, *Porterella*, *Diastatea*, *Hypsela*, *Hippobroma*, and *Lithotoma* had only one representative in each genus. *Pratia borneensis* Hemsl. (Hemsley 1886), *Lobelia physaloides* A.Cunn. (Cunningham 1838), Lobelia heterophylla subsp. heterophylla, and some members of the *Lobelia* § *Delostemon* (E.Wimm.) J.Murata (Murata 1995) formed the basal group of Lobelioideae with a BS value of 83 (Fig. 1).

Lobelioideae originated from Africa and this corroborated Knox et al. (2006) and Knox and Li (2017). Nemacladoideae forms a sister to the Lobelioideae group. This group is endemic to North America. However, their position and endemicity in North America do not affect the African origin of Lobelioideae (Fig. 2). Knox et al. (2006) stated that the removal and/or inclusion of *Cyphia* clade (Endemic to Africa) as the sister to Lobelioideae would not interfere with the African origin of this subfamily, and this corroborated with our biogeographic results. Besides, it is also evident that multiple dispersal events occurred in this subfamily. The basal group consisted of species with their ancestral region in Australasia and Africa. The African group nested some species from Asia, America, and the Pacific Islands. This depicted possibilities of long-distance dispersal and diversification events in some species.

The following is a discussion of specific genera within the Lobelioideae subfamily. The order of the discussion is according to the positioning of the genera in the phylogram, starting from the basal position (Fig. 1). ***Pratia*** is polyphyletic. *Pratia borneensis* is a sister (BS = 83) to a clade formed by *Lobelia physaloides*, L heterophylla subsp. heterophylla and seven members from the *Lobelia* § *Delostemon*. *Pratia angulata* Hook.f. (Hooker 1844), forms a clade with *Lobelia chinensis* Lour. (de Loureiro 1790) with a BS value of 86. *Pratia nummularia* A.Braun & Asch. (Braun 1861), on the other hand forms a clade with *Lobelia angulata* with a BS value of 100, while *Pratia purpurascens* (R.Br.) E.Wimm. (Wimmer 1953), forms a clade with *Lobelia arnhemiaca* E.Wimm. (Wimmer 1948) with a BS value of 61 (Fig. 1). Biogeography: Murray et al. (2004) described New Zealand as the ancestral region of *Pratia*. This was also echoed by Knox et al. (2008b) in their work on the phylogenetic position of *Lobelia glaberrima* Heenan (Heenan et al. 2008), in New Zealand. Our analysis placed this genus in Australasia as the ancestral region (Fig. 2).

***Grammatotheca*** has only one species, *Grammatotheca bergiana* C.Presl (Presl 1836). It is nested within some members of the *Lobelia* § *Delostemon* with a BS value of 62 (Fig. 1). Our results confirmed that of Antonelli (2009) and Chen et al. (2016). Biogeography: Knox et al. (2006) indicated South Africa as the origin of this genus. Chen et al. (2016) also placed *Grammatotheca* in Africa. This genus is embedded within *Lobelia* § *Delostemon* clade which has its ancestral region in Africa. Knox et al. (2006) stated that holopogonoid *Lobelia* gave rise to this particular genus which diversified in South Africa and was later introduced to Australia via hay shipped with cattle from South Africa. Our results corroborate the above-mentioned studies and placed this genus in Africa as its ancestral area (Fig. 2).

***Monopsis*** forms a monophyletic group with a BS value of 91. It forms a clade with members of the *Lobelia* § *Delostemon* with a BS value of 100 (Fig. 1). This result corroborates that of Chen et al. (2016). Biogeography: Phillipson (1989) indicated Cape Province, South Africa as the ancestral region of *Monopsis*. Knox et al. (2006) also indicated South Africa as the ancestral region of this genus. Our result placed this genus in Africa as its ancestral region (Fig. 2).

***Wimmerella*** forms a clade with a BS value of 100. They form a sister to *Lobelia anceps* L.f. (Linnaeus 1782) with a BS value of 100 (Fig. 1). This result corroborates that of Knox and Li (2017) and Chen et al. (2016). Biogeography: Knox et al. (2006) placed this genus in Western Cape, South Africa as its ancestral region. Chen et al. (2016) and Knox and Li (2017) corroborated Knox et al. (2006) results. Our analysis corroborates the above-mentioned studies and placed this particular genus in Africa (Fig. 2).

***Delissea*** and ***Brighamia*** form a clade with a BS value of 86, a result similar to that of Murata (1995), Antonelli (2008), Givnish et al. (2009), Chen et al. (2016), and Knox and Li (2017). Biogeography: These genera were placed in Kaua’i or some older island as their ancestral region (Givnish et al. 1994, 2004, 2009; Givnish 1995). Our results place these genera in the Pacific Islands as their ancestral area which corroborates both Givnish et al. (2004) and Knox and Li (2017).

***Trematolobelia*** forms a clade with a BS value of 99 (Chen et al. 2016) (Fig. 1). This clade forms a sister to *Lobelia kauaensis* (A.Gray) A.Heller (Heller 1897), with a BS value of 90. Biogeography: Givnish (1998) placed *Trematolobelia* on Kaua`i as its ancestral area. Givnish et al. (2009) indicated the Hawaiian archipelago as the ancestral region of this particular genus. Our analysis placed this genus in the Pacific Islands as its ancestral area which corroborates the above-mentioned studies (Fig. 2).

***Apetahia*** and ***Sclerotheca*** form a clade with a BS value of 64. This result confirms that of Givnish et al. (2009) and Chen et al. (2016). We sampled only three *Apetahia* and eight *Sclerotheca* species. The low BS value might have been a result of incomplete sampling in these two genera. *Sclerotheca margaretae* F.Br. (Brown 1935), *Sclerotheca viridiflora* Cheeseman (Cheeseman 1903), *Sclerotheca oreades* E.Wimm. (Wimmer 1948), *Sclerotheca arborea* DC. (Candolle 1839), *Sclerotheca forsteri* Drake (Del Castillo 1892), and *Sclerotheca magdalenae* J.Florence (Florence 1996) form a clade with a BS value of 60. Biogeography: Chen et al. (2016) placed these genera in French Polynesia as their ancestral area. Knox and Li (2017) indicated the ancestral location of *Apetahia longistigmata* (F.Br.) E.Wimm. (Wimmer 1948) to be in Marquesas and *S. viridiflora* to be in Rarotonga, both in the South Pacific Islands. Our results placed the genera in the Pacific Islands as their ancestral area which concur with the above-mentioned studies.

***Cyanea*** forms a paraphyletic group (Fig. 1). This result corroborates with Antonelli (2009), Chen et al. (2016), and Hunter (2018). *Cyanea aspleniifolia* Hillebr. (Hillebrand 1888), *Cyanea koolauensis* Lammers, Givnish and Sytsma (Lammers et al. 1993), and *Cyanea fissa* Hillebr. (Hillebrand 1888) form a clade with a BS value of 98 that is sister to *Clermontia* (BS=77) (Fig. 1). ***Clermontia*** forms a clade (Givnish et al. 2009, 2013; Chen et al. 2016) with a BS value of 90 (Fig. 1). More broadly, Hunter (2018) used phylogenomic data from hundreds of single-copy nuclear genes and whole plastomes to infer that most of *Clermontia* are sister to the purple-fruited clade of *Cyanea* (see Givnish et al. 1994; Givnish 1995), with the orange-fruited clade of *Cyanea* sister to both. Biogeography: Givnish et al. (1994, 2009, 2013) placed the origins of both *Clermontia* and *Cyanea* on Kaua`i or some older island. Chen et al. (2016) and Knox and Li (2017) placed these two genera in the Hawaiian Islands as their ancestral region. Our results placed the genera in the Pacific Islands as their ancestral area, corroborating the above-mentioned studies (Fig. 2).

***Solenopsis*** is monophyletic with a BS=100. *Lobelia urens* L. (Linnaeus 1753) (*Lobelia* § *Stenotium* (C.Presl) Lammers (Lammers 2011)) formed a sister to *Solenopsis* with the BS value of 87 (Fig. 1). Our results corroborate that of Knox and Li (2017). Biogeography: Crespo et al. (1998) indicated the Mediterranean as the ancestral region of this genus. Knox and Li (2017) also indicated the Mediterranean as the ancestral area for *Solenopsis*. Our result corroborates that of Crespo et al. (1998) and Knox and Li (2017) and places this genus in the Mediterranean region as its ancestral region (Fig. 2).

***Downingia*** formed a monophyletic clade with a BS value of 96. ***Porterella*** is sister to *Downingia* with a BS value of 98. This result corroborates Chen et al. (2016). ***Legenere****limosa* (Greene) McVaugh (McVaugh 1943a), and *Legenere valdiviana* (Phil.) E.Wimm. (Wimmer 1953) form a clade (BS=100), which is a sister to *Downingia* and *Porterella*. ***Palmerella*** forms a sister to *Downingia*, *Porterella*, and *Legenere* with a BS of 90 (Fig. 1). Biogeography: McVaugh (1941) indicated western North America as the ancestral area of *Downingia*. Schultheis (2001a, b) corroborated McVaugh (1941). Chen et al. (2016) placed *Downingia*, *Porterella*, and *Legenere* in North America. Our analysis places the genera in North America as their ancestral region (Fig. 2).

***Diastatea*** is clustered with *Lobelia nana* Kunth (Kunth et al. 1976) with a BS value of 62 (Fig. 1). However, our phylogenetic results contradicted that of Chen et al. (2016) that appeared to form a clade with *Solenopsis*. *Diastatea* was differentiated from genus *Lobelia* by two main features: a superior ovary, and a persistent corolla lacking a dorsal fissure (McVaugh 1940). Albeit these characters have been used to separate the two genera, some species in the *Lobelia* § *Stenotium* (featured by partially inferior to the superior ovary) possess the same characteristics e.g. *Lobelia xalapensis* Kunth (Kunth et al. 1976), *L. nana*, and *Lobelia diastateoides* McVaugh (McVaugh 1940). Biogeography: Knox et al. (2008a) placed the genus in South America as the ancestral area. Chen et al. (2016) also indicated America as the ancestral area of *Diastatea*. Our biogeographic results corroborate both Knox et al. (2008a) and Chen et al. (2016) and place the genus in South America as its ancestral area.

***Hippobroma*** is monotypic and is nested within members of the *Lobelia* § *Tylomium* (Fig. 1). This result corroborates Chen et al. (2016). Biogeography: Knox and Li (2017) indicated Mexico as the ancestral region of *Hippobroma*. The ancestral area of § *Tylomium*, which nestles this genus, is in North America (Lammers 2011). This genus might have resulted following morphological diversification. This corroborates Chen et al. (2016) that placed the genus in Central America. Our result places the genus in Central America as its ancestral area which corroborates the above-mentioned studies.

***Isotoma*** is polyphyletic. *Isotoma hypocrateriformis* Druce (Druce 1917), is sister to *P. angulata*, *P. nummularia*, *P. purpurascens*, ***Hypsela***, ***Lithotoma***, and some *Lobelia* species belonging to the *Lobelia* § *Hypsela* (C.Presl) Lammers (Lammers 2011), with a BS value of 96. All these genera are from the Australasian region except for *Lobelia loochooensis* Koidz. (Koidzumi 1929), and *L. chinensis* that are from Southeast Asia and *Lobelia oligophylla* (Wedd.) Lammers (Lammers 1999), from South America. *Isotoma tridens* (E.Wimm.) Lammers (Lammers 1999), forms a clade with *Hypsela tridens* E.Wimm. (Wimmer 1943) with a BS value of 100. *Isotoma fluviatilis* F.Muell. ex Benth. (Bentham and Mueller 1869) is sister to *L. chinensis* and *P. angulata* with a BS value of 81. *Isotoma axillaris* Lindl. (Lindley 1826) forms a clade with *Lobelia petraea* with a BS value of 91 (Fig. 1). According to Givnish et al. (2009), *Isotoma* formed a sister to the tropical American taxa. Our results corroborate that of Chen et al. (2016). Biogeography: Bussell et al. (2002) and Chen et al. (2016) placed *Isotoma* in Australia as its ancestral region. Knox and Li (2017) placed *Hypsela* and *Lithotoma* in Australasia as their ancestral region. Our results place *Isotoma*, *Lithotoma*, and *Hypsela* in Australasia as their ancestral area which corresponds to the above-mentioned studies (Fig. 2).

***Lysipomia****pumila* (Wedd.) E.Wimm. (Wimmer 1937), *Lysipomia cuspidata* McVaugh (McVaugh 1955), and *Lysipomia sphagnophila* Griseb. (Lechler 1857) forms a clade with a BS value of 100 (Fig. 1). This result is consistent with that of Antonelli (2008) and Chen et al. (2016). Biogeography: McVaugh (1955), in his revision of *Lysipomia*, indicated South America as the ancestral area of this particular genus. Knox and Li (2017) stated diversification in S. America generated *Lysipomia*. Our results placed *Lysipomia* in South America as its ancestral region which corroborates McVaugh (1955) (Fig. 2).

***Siphocampylus*** and ***Centropogon*** are polyphyletic and intercalates with each other, albeit their statistical support values are low (<50) (Fig. 1). *Centropogon dianae* Lammers (Lammers 1998), *Siphocampylus sparsipilus* E.Wimm. (Wimmer 1924), *Centropogon brittonianus* Zahlbr. (Zahlbruckner 1897), *Centropogon gloriosus* Zahlbr. (Zahlbruckner 1897), *Centropogon simulans* Lammers (Lammers 1998), *Centropogon peruvianus* (E.Wimm.) McVaugh (McVaugh 1949a), *Centropogon luteynii* Wilbur (Wilbur 1977), *Centropogon costaricae* (Vatke) McVaugh (McVaugh 1943a), and *Siphocampylus manettiiflorus* Hook. (Hooker 1848) forms an early clade with the members of the *Lobelia* § *Tylomium*. *Centropogon nigricans* Zahlbr. (Zahlbruckner 1915) is sister to the *Burmeistera* clade (Fig. 1). ***Burmeistera*** forms a clade with a low support value (BS=43) (Fig. 1). The low BS values for *Burmeistera* and *Siphocampylus* may be due to inadequate taxon sampling (Uribe-Convers et al. 2017). Biogeography: The ancestral areas for *Centropogon*, *Burmeistera*, and *Siphocampylus* are in South America (Antonelli 2009; Knox and Li 2017; Uribe-Convers et al. 2017). Our analysis placed these genera in S. America which corroborates the above-mentioned (Fig. 2).

### Sections within genus *Lobelia*

*Lobelia* is the ‘core genus’ among members of the Lobelioideae group (Knox et al. 2006). Lammers (2011) classified this genus into eighteen sections based on morphological characteristics. Our analysis included fifteen out of the eighteen sections: four monophyletic, five polyphyletic, one paraphyletic and five had only one representative each. The unsampled sections were *Speirema* (Hook.f. and Thomson) Lammers (Lammers 2010), *Trimeris* (C.Presl) A.DC. (Candolle 1839), and *Plagiobotrys* Lammers (Lammers 2010), (Table 2).

***Lobelia*** § ***Holopogon*** Benth. (Bentham and Mueller 1869). This section had only one out of fourteen species (Lammers 2011) sampled in a combined plastid dataset, that is, Lobelia heterophylla subsp. heterophylla. It occurred at the basal position of the phylogram and formed a clade with *L. physaloides* with a BS value of 92 (Fig. 1). Our analysis corroborated that of Antonelli (2008) and Knox et al. (2006). Biogeography: Lammers (2011) indicated Australia as the ancestral area of L heterophylla subsp. heterophylla. Our biogeographic results corroborate the above-mentioned studies and place this species in Australasia as its ancestral area (Fig. 2).

***Lobelia*** § ***Colensoa*** (Hook.f.) J.Murata (Murata 1995). Only one species was sampled in the combined plastid dataset. According to Lammers (2011), this section is monotypic. *Lobelia physaloides* forms a sister clade with L heterophylla subsp. heterophylla (§ *Holopogon*) with a bootstrap value of 92 (Fig. 1). In the ITS phylogram, it is embedded between the *Lobelia* § *Stenotium* and *Lobelia* § *Delostemon*. Biogeography: Lammers (2011) placed this section in New Zealand’s North Island as the ancestral location. Our results place this section in Australasia as its ancestral region which corroborates Lammers (2011).

***Lobelia*** § ***Delostemon***. This section is paraphyletic. We sampled fourteen out of forty-four species recorded by Lammers (2011) in our combined plastid dataset. *Lobelia baumannii* Engl. (Engler 1894), *Lobelia hartlaubi* Buchenau (Buchenau 1881), *Lobelia malowensis* E.Wimm. (Wimmer 1948), *Lobelia patula* L.f. (Linnaeus 1782), *Lobelia neglecta* Roem. and Schult. (Roemer and Schultes 1819), *Lobelia coronopifolia* L. (Linnaeus 1753), *Lobelia holstii* Engl. (Engler 1894), and *Lobelia tomentosa* L.f. (Linnaeus 1782) form a clade with a BS = 84. *Lobelia thermalis* Thunb. (Thunberg 1794) is sister to a clade of *Lobelia aquatica* Cham. (Chamisso 1833) and *Lobelia zeylanica* L. (Linnaeus 1753) with a BS value of 88. However, this clade is intercalated by *Grammatotheca*. *Lobelia capillifolia* A.DC. (Candolle 1839) and *Lobelia linearis* Thunb. (Thunberg 1794) form a clade with a BS value of 97 and is sister to *Monopsis* with a BS value of 100 (Fig. 1). Our result corroborates that of Chen et al. (2016), Antonelli (2008), and Knox et al. (2006). Antonelli (2008) clustered *Grammatotheca* and *L. aquatica* together and further indicated the similarities between them, that is, both are slender annual herbs and have smaller dorsal corolla lobes. These similarities are also observed in *Monopsis debilis* (L.f.) C.Presl (Presl 1836), (Phillipson 1986). Lammers (2011) described plants in the *Lobelia* § *Delostemon* as perennial with prostrate, decumbent, and ascending stems, sessile or petiolate leaves, bilabiate corolla, and capsular fruit. These features are also found in *Monopsis alba* Phillipson (Phillipson 1986), *Monopsis simplex* (L.) E.Wimm. (Wimmer 1948), and *Monopsis stellarioides* Urb. (Urban 1881), (Phillipson 1986). Antonelli (2008) suggested that if a cladistic approach of classification were to be observed strictly, then *Grammatotheca* and *Monopsis* would have been placed under this section. A suggestion that is highly supported by our analysis. Biogeography: Our analysis indicates Africa as the ancestral area of this section. However, it embeds two species; *L. aquatica* and *L. zeylanica* which were placed in South America and Southeast Asia respectively. Our results corroborated that of Antonelli (2009) and Lammers (2011).

***Lobelia*** § ***Mezleriopsis*** Lammers (Lammers 2011). This section had only one out of seven species (Lammers 2011) sampled in the combined plastid dataset. *Lobelia sonderiana* (Kuntze) Lammers (Lammers 1999) forms a sister to the remaining members of the Lobelioideae group except for *Grammatotheca*, *Monopsis*, *Lobelia* § *Delostemon*, *Lobelia* § *Colensoa*, *Lobelia* § *Holopogon*, and *P. borneensis* (BS = 56) (Fig. 1). This result corroborates Antonelli (2008). Biogeography: Lammers (2011) indicated the ancestral area of this section to be in South Africa with *L. sonderiana* extending up to Kenya. This corroborates with our results and places Africa as the ancestral region of this section (Fig. 2).

***Lobelia*** § ***Stenotium***. We sampled ten species out of a hundred and forty-four proposed by Lammers (2011) in our combined plastid dataset. This section is polyphyletic. *Lobelia laxa* McOwan (MacOwan 1890), *L. erinus*, *Lobelia graniticola* E.Wimm. (Wimmer 1948), and *Lobelia fervens* Thunb. (Thunberg 1794) formed a clade with a BS value of 100. *Lobelia anceps* (BS = 100) is sister to a clade of *Wimmerella pygmaea* (Thunb.) Serra M.B. Crespo and Lammers (Serra et al 1999) and *Wimmerella hederacea* (Sond.) Serra and Lammers (Serra et al 1999). *Lobelia muscoides* Cham. (Chamisso 1833) forms a clade with *Lobelia jasionoides* (A.DC.) E.Wimm. (Wimmer 1943) with a BS value of 100. *Lobelia urens* forms a sister to *Solenopsis* with a BS value of 87. *Lobelia nana* and *Diastatea* form a clade with a BS value of 62 (Fig. 1). According to our phylogenetic analysis, this section appears to be polyphyletic, a suggestion that corroborates Antonelli (2008) and Knox et al. (2006). More so, *Solenopsis*, just like *Wimmerella*, has corolla completely fused (Knox et al. 2006). Lammers (2011) suggested the inclusion of *Wimmerella* in this section. A suggestion that is well supported by our phylogenetic analysis. Biogeography: Our analysis placed the ancestral area of this section in Africa. However, *L. nana* and *L. xalapensis* have their ancestral areas in South America while *L. urens* has its ancestral region in the Mediterranean. Our results corroborated that of Lammers (2011).

***Lobelia*** § ***Jasionopsis*** Lammers (Lammers 2011). Only one sample was analyzed in our combined plastid dataset. This section is monotypic. The sampled species included *L. jasionoides* which form a clade with *L. muscoides* (§ *Stenotium*) with a BS value of 100 (Fig. 1). This corroborates Knox and Li (2017). Biogeography: Lammers (2011) described this species as endemic to the Cape provinces of South Africa. Chen et al. (2016) placed it in Africa as its ancestral area. Knox and Li (2017) corroborated both analyses. Our result places Africa as its ancestral region (Fig. 2) which corroborates the above-mentioned studies. The close relationship between *L. jasionoides* and *L. muscoides*, both statistically (BS = 100) and geographically (both in S. Africa), suggest a biphyletic nature of this section and/or the inclusion of *L. jasionoides* in *Lobelia* § *Stenotium*.

***Lobelia*** § ***Rhynchopetalum*** (Giant Lobelioids/Rosettes). We sampled forty-eight out of the sixty-one species (Lammers 2011) in our combined plastid dataset. This section is polyphyletic. *Dialypetalum*, *Brighamia*, *Delissea*, *Trematolobelia*, and *Lobelia* § *Revolutella* E.Wimm. (Wimmer 1948) are all embedded within this section. *Lobelia sessilifolia* Lamb. (Lambert 1811) is sister to the members of this section however with a low support value of 26 (Fig. 1). This corroborates Chen et al. (2016), Crowl et al. (2016), and Knox and Li (2017) that the ancestor to giants lobelioids might have its ancestral region in S.E Asia. Biogeography: Lammers (2011) described this section as almost pantropical with species in three disjunct areas, that is, Southeast Asia, tropical Africa, and South America. According to our results, species with S.E. Asia as their ancestral region formed the basal group of this section. *Lobelia sessilifolia* forms a sister to the members of this section. Our analysis placed this taxon in S.E. Asia as its ancestral area, corroborating Chen et al. (2016) that the ancestor of the giant lobelioids could have been from S.E Asia. Our result corroborates that of Lammers (2011), Chen et al. (2016), and Knox and Li (2017) (Fig. 2).

***Lobelia*** § ***Revolutella***. We sampled three species out of nine (Lammers 2011): *Lobelia niihauensis* St.John (John 1931), *Lobelia yuccoides* Hillebr. (Hillebrand 1888), and *Lobelia hypoleuca* Hillebr. (Hillebrand 1888). They form a clade with a BS value of 100 (Fig. 1). This corroborates Givnish’s (1998), Antonelli’s (2008), Givnish et al.’s (2009), and Chen et al.’s (2016) results. Biogeography: Lammers (2011) and Chen et al. (2016) indicated the Hawaii archipelago as the ancestral area of sampled members of this section. A more detailed phylogenomic analysis by Hunter (2018) placed the origin of § *Revolutella* in Kaua`i. Our analysis corroborates the above-mentioned studies and places this section in the Pacific Islands as its ancestral region (Fig. 2).

***Lobelia*** § ***Galeatella*** E.Wimm. (Wimmer 1948). In this section, we sampled one species, that is, *L. kauaensis*. Lammers (2011) included five species in this section, however, *L. kauaensis* was not amongst those included. Lammers (2007b) indicated that this species is a hybrid of natural taxa (nothotaxon). It forms a sister to *Trematolobelia* with a BS value of 90. Our results corroborate with that of Chen et al. (2016). Biogeography: Lammers (2007b, 2011) and Hunter (2018) stated that this section has its ancestral region in the Hawaiian archipelago. This corroborates with our results as it places this section in the Pacific Islands as its ancestral region.

***Lobelia*** § ***Lobelia***. Eleven out of twenty-two species (Lammers 2011) were sampled in our combined plastid dataset. This section is monophyletic. *Lobelia inflata* L. (Linnaeus 1753), and *Lobelia kalmii* L. (Linnaeus 1753) form a clade with a BS value of 68 and form sister to members of this section with a BS value of 96 (Fig. 1). This result corroborates Antonelli (2008). Biogeography: Lammers (2011) indicated North America as the ancestral location of this section. Our analysis concurs with Lammers (2011) and places North America as the ancestral area of this section (Fig. 2).

***Lobelia*** § ***Cryptostemon*** (E.Wimm.) J.Murata (Murata 1995). We sampled two out of nine species included by Lammers (2011) in this section. The combined plastid dataset included *Lobelia irasuensis* Planch. & Oerst. (Planchon and Oersted 1857), whereas *Lobelia fenestralis* Cav. (Cavanilles 1791) was included in the ITS dataset. *Lobelia irasuensis* forms a clade with *Lobelia divaricata* Hook. and Arn. (Hooker et al. 1838) with a BS=96 (Fig. 1) while *L. fenestralis* forms a clade with *Lobelia laxiflora* Kunth (Kunth and Bonpland 1820) with a BS = 59. Biogeography: Chen et al. (2016) placed *L. irasuensis* in Central America as its ancestral region, which corroborated Antonelli (2009). Our analysis places this section in Central America as the ancestral region which corroborates with the above-mentioned studies (Fig. 2).

***Lobelia*** § ***Homochilus*** DC. (Candolle 1839). We sampled two out of five species (Lammers 2011). *Lobelia laxiflora* Kunth (Kunth and Bonpland 1820) and *Lobelia aguana* E.Wimm. (Wimmer 1935) form a clade with a BS value of 48 (Fig. 1). Biogeography: Givnish et al. (2009) and Chen et al. (2016) indicated the ancestral area of these two species to be in Central America. Our results corroborate the above-mentioned and places this section in Central America.

***Lobelia*** § ***Tylomium***. Six out of thirty-eight species were sampled. This section is paraphyletic. *Hippobroma longiflora* (L.) G.Don (Don 1834) is nested within this section. *Lobelia portoricensis* Urb. (Urban et al 1899), *Lobelia kraussii* Graham (Graham 1830), *Lobelia martagon* Hitchc. (Hitchcock 1893), *Lobelia stricta* Sw. (Swartz 1788), and *Lobelia vivaldii* form a clade (Fig. 1). Biogeography: *Lobelia assurgens* L. (Linnaeus 1759), *L. portoricensis*, *L. martagon*, and *L vivaldii* were placed in the Greater Antilles as their ancestral region while *L kraussi* and *L. stricta* were placed in the Lesser Antilles as their ancestral region (Lammers 2011). Chen et al. (2016) indicated Central America as their ancestral area which corroborated our results.

***Lobelia*** § ***Hypsela***. Thirteen out of forty-three species within this section were sampled. This section is polyphyletic. It is intercalated with *Isotoma*, *Hypsela*, *Pratia*, and *Lithotoma* species. *Lobelia fugax* Heenan, Courtney & P.N.Johnson (Heenan et al 2008), *Lobelia ionantha* Heenan (Heenan et al 2008), *Lobelia fatiscens* Heenan (Heenan et al 2008), and *Lobelia carens* Heenan (Heenan et al 2008) form a clade with a BS value 98. *Lobelia roughii* Hook.f. (Hooker 1864), *Lobelia linnaeoides* Petrie (Petrie 1890), *Lobelia macrodon* (Hook.f.) Lammers (Lammers 1998), *Lobelia glaberrima* Heenan (Heenan et al 2008), and *L. oligophylla* also form a clade with a BS value of 70. *Lobelia arnhemiaca* forms a clade with *P. purpurascens* with a BS value of 61. *Lobelia oligophylla* is sister to *L. angulata*, *L. roughii*, *L. macrodon*, *L. glaberrima*, and *L. linnaeoides* (Fig. 1). This result corroborates that of Antonelli (2008). In the ITS region dataset, *Pratia*, *Isotoma*, *Hypsela*, and *L. chinensis* form a clade with a BS=100. Lammers (2011) indicated the chromosome number of this section as 2*n*=12, 14, 28, 42, 56, 70, 77, 84, 91 and 140. *Pratia* also shows these same chromosome number variations, consistent with it being an exemplar of interspecific hybridization. *Pratia angulata* is 2*n*=70 while *P. perpusilla* is 2*n*=42, the hybrids between these two species have 2*n*=77, 91, and 140 chromosome numbers reported (Murray et al. 2004). According to Knox et al. (2008b), *Isotoma* was distinguished by floral fusion with adnate filaments, *Pratia* on the other hand was classified using berry fruits, and *Hypsela* was differentiated by having both floral fusion and berry fruits. These features are similar to those used by Lammers (2011) used to describe the *Lobelia* § *Hypsela*. More so, Lammers (2011) suggested the inclusion of *Isotoma* in this section, a suggestion that is well supported by our phylogenetic analysis. Our analysis proposes the inclusion of *Hypsela*, *Pratia*, and Lithotoma in this section too. Biogeography: Lammers (2011) described this section as Amphi-pacific with a majority of the species in the southern hemisphere. Our analysis placed this section in Australasia as their ancestral region although *L. loochooensis* and *L. chinensis* were placed in Asia as their ancestral region. *Lobelia oligophylla*, on the other hand, has its ancestral region in South America. Our analysis corroborated that of Lammers (2011) (Fig. 2).

***Lobelia*** § ***Tupa*** (G.Don) Benth. (Bentham 1876). We sampled all four members of this section in our combined plastid dataset. The sampled species included *Lobelia polyphylla* Hook. & Arn. (Hooker and Arnott 1830), *Lobelia bridgesii* Hook. & Arn. (Hooker and Arnott 1830), *Lobelia tupa* L. (Linnaeus 1753), and *Lobelia excelsa* Bonpl. (Bonpland et al. 1816), and form a monophyletic group with a BS value of 99 (Fig. 1). So far, this corroborates Lammers and Hensold (1992) and Antonelli (2008) that species belonging to this section might be monophyletic due to the uniform occurrence of an unusual chromosome number (2*n*=42). Biogeography: The ancestral area of this section is Chile (Lammers 2011). Chen et al. (2016) corroborated Lammers’s (2011) results. Our result is consistent with both of them and places South America as the ancestral region of this section.

## Conclusion

In this study, we conducted a literature review and phylogenetic analyses on Lobelioideae. We found that previous studies have currently reached an agreement on the southern African origin of Lobelioideae, herbaceous habit, and Asian origin of giant lobelioids, and lastly, the convergent evolution of giant rosette lobelioids. We also found that several genera, such as *Lobelia*, are polyphyletic and their systematics is particularly frustrating, which calls for further reappraisals using both morphological and molecular data. More so, taxon sampling and sequencing of some genera such as *Centropogon*, *Burmeistera*, *Siphocampylus*, and *Clermontia* are quite minimal. The phylogenetic analyses in this paper were based primarily on 18 plastid loci; the resolution and support provided by ITS were weak. Future advances in Lobelioideae phylogenetics should include phylogenomic approaches based on hundreds of single-copy nuclear genes and flanking regions, and direct assessment of possible hybridization, incomplete lineage sorting, or other forms of reticulate evolution, to investigate extensively the classification of Lobelioideae.

## Supplementary materials

Supplementary material 1 Lobelioideae data matrix Data type sequence data Explanation note Sequence GenBank ID and voucher information of all the sequences used in the study. Sheet 1. Linked data table for sequences and voucher information used in the study. heet 2. Lobelioideae taxa in the combined plastid dataset. Sheet 3. References of sequences used in the study. https://binary.pensoft.net/file/516735This dataset is made available under the Open Database License (http://opendatacommons.org/licenses/odbl/1.0/). The Open Database License (ODbL) is a license agreement intended to allow users to freely share, modify, and use this Dataset while maintaining this same freedom for others, provided that the original source and author(s) are credited.Samuel Paul Kagame, Andrew W. Gichira, Lingyun Chen, Qingfeng Wang

Supplementary material 2 Figure S1. Phylogeny of Campanulaceae family with bootstrap values using combined cp dataset Data type Phylogenetic Explanation note This file contains the bootstrap values for the whole campanulaceae family generated from combined chloroplast dataset using maximum likelihood analysis. https://binary.pensoft.net/file/516736This dataset is made available under the Open Database License (http://opendatacommons.org/licenses/odbl/1.0/). The Open Database License (ODbL) is a license agreement intended to allow users to freely share, modify, and use this Dataset while maintaining this same freedom for others, provided that the original source and author(s) are credited.Samuel Paul Kagame, Andrew W. Gichira, Lingyun Chen, Qingfeng Wang

Supplementary material 3 Figure S2. Phylogeny of Campanulaceae family with bootstrap values using ITS dataset Data type Phylogenetic Explanation note Phylogenetic tree of whole campanulaceae family with boostrap values generated from Internal Transcribed Spacer (ITS) regions using maximum likelihood analysis. https://binary.pensoft.net/file/516737This dataset is made available under the Open Database License (http://opendatacommons.org/licenses/odbl/1.0/). The Open Database License (ODbL) is a license agreement intended to allow users to freely share, modify, and use this Dataset while maintaining this same freedom for others, provided that the original source and author(s) are credited.Samuel Paul Kagame, Andrew W. Gichira, Lingyun Chen, Qingfeng Wang

Supplementary material 4 Figure S3. Phylogeny, genera and bootstrap values of Lobelioideae using ITS dataset Data type Phylogenetic image Explanation note Phylogenetic tree image indicating the genera classification and bootstrap values of lobelioideae subfamily trimmed from ITS campanulaceae tree. https://binary.pensoft.net/file/516738This dataset is made available under the Open Database License (http://opendatacommons.org/licenses/odbl/1.0/). The Open Database License (ODbL) is a license agreement intended to allow users to freely share, modify, and use this Dataset while maintaining this same freedom for others, provided that the original source and author(s) are credited.Samuel Paul Kagame, Andrew W. Gichira, Lingyun Chen, Qingfeng Wang

Supplementary material 5 Figure S4. Phylogeny of Lobelioideae with bootstrap values using combined plastid dataset Data type Phylogenetic Explanation note Phylogenetic tree of lobelioideae subfamily trimmed from combined plastid campanulaceae tree. https://binary.pensoft.net/file/516739This dataset is made available under the Open Database License (http://opendatacommons.org/licenses/odbl/1.0/). The Open Database License (ODbL) is a license agreement intended to allow users to freely share, modify, and use this Dataset while maintaining this same freedom for others, provided that the original source and author(s) are credited.Samuel Paul Kagame, Andrew W. Gichira, Lingyun Chen, Qingfeng Wang
