# Supplementary material for: Systematics of Lobelioideae (Campanulaceae): review, phylogenetic and biogeographic analyses
Source: PhytoKeys. 2021 Mar 5;174:13–45. doi: 10.3897/phytokeys.174.59555 (PMC7954781; doi:10.3897/phytokeys.174.59555)
Supplement: Supplementary material 4 — Figure S3. Phylogeny, genera and bootstrap values of Lobelioideae using ITS dataset [file phytokeys-174-013-s004.pdf]

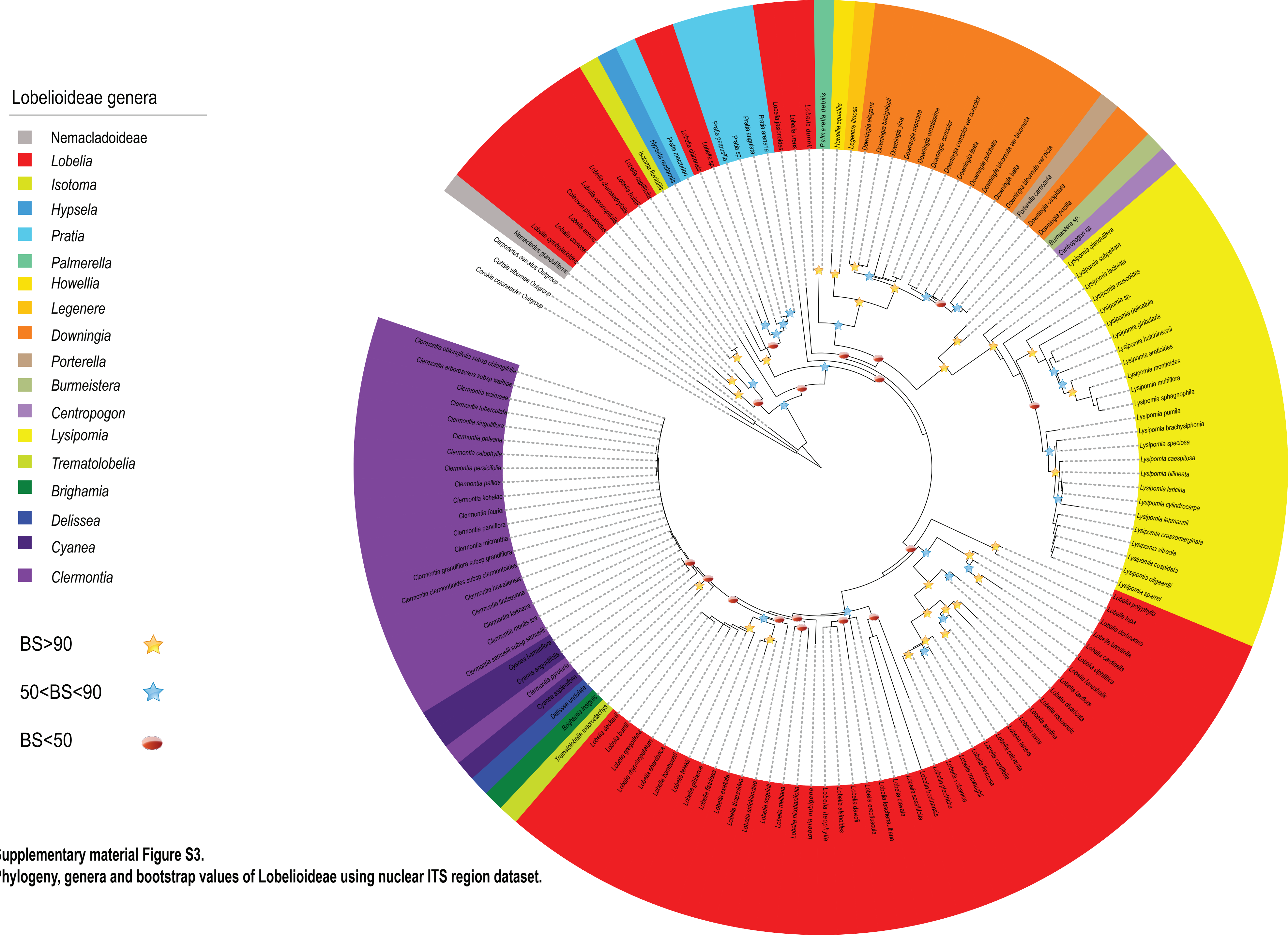

Supplementary material Figure S3.
Phylogeny, genera and bootstrap values of Lobelioideae using nuclear ITS region dataset.
