# Supplementary material for: Systematics of Lobelioideae (Campanulaceae): review, phylogenetic and biogeographic analyses
Source: PhytoKeys. 2021 Mar 5;174:13–45. doi: 10.3897/phytokeys.174.59555 (PMC7954781; doi:10.3897/phytokeys.174.59555)
Supplement: Supplementary material 5 — Figure S4. Phylogeny of Lobelioideae with bootstrap values using combined plastid dataset [file phytokeys-174-013-s005.pdf]

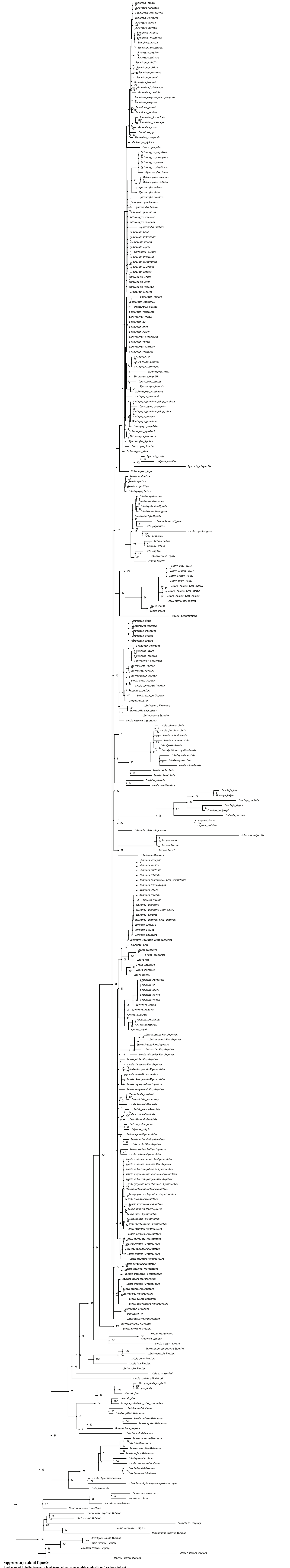

Supplementary material Figure S4.
Phylogeny of Lobelioideae with bootstrap values using combined plastid (cp) regions dataset.
