# Supplementary material for: Systematics of Lobelioideae (Campanulaceae): review, phylogenetic and biogeographic analyses
Source: PhytoKeys. 2021 Mar 5;174:13–45. doi: 10.3897/phytokeys.174.59555 (PMC7954781; doi:10.3897/phytokeys.174.59555)
Supplement: Supplementary material 2 — Figure S1. Phylogeny of Campanulaceae family with bootstrap values using combined cp dataset [file phytokeys-174-013-s002.pdf]

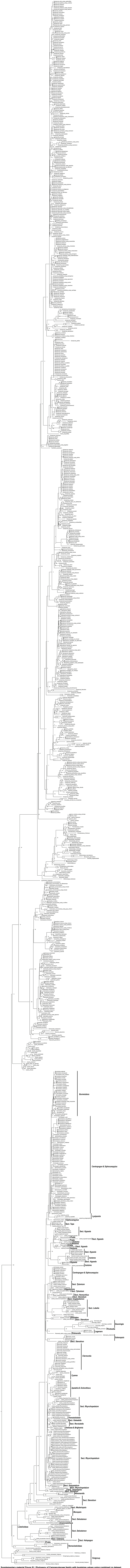

Supplementary material Figure S1. Phylogeny of Campanulaceae family with bootstrap values using combined cp dataset.
